# PDNAsite: Identification of DNA-binding Site from Protein Sequence by Incorporating Spatial and Sequence Context

**DOI:** 10.1038/srep27653

**Published:** 2016-06-10

**Authors:** Jiyun Zhou, Ruifeng Xu, Yulan He, Qin Lu, Hongpeng Wang, Bing Kong

**Affiliations:** 1 School of Computer Science and Technology, Harbin Institute of Technology Shenzhen Graduate School, Shenzhen, Guangdong, China; 2 Department of Computing, the Hong Kong Polytechnic University, Hong Kong; 3 Shenzhen Engineering Laboratory of Performance Robots at Digital Stage, Shenzhen Graduate School, Harbin Institute of Technology, Shenzhen, China; 4 School of Engineering and Applied Science, Aston University, UK

## Abstract

Protein-DNA interactions are involved in many fundamental biological processes essential for cellular function. Most of the existing computational approaches employed only the sequence context of the target residue for its prediction. In the present study, for each target residue, we applied both the spatial context and the sequence context to construct the feature space. Subsequently, Latent Semantic Analysis (LSA) was applied to remove the redundancies in the feature space. Finally, a predictor (PDNAsite) was developed through the integration of the support vector machines (SVM) classifier and ensemble learning. Results on the PDNA-62 and the PDNA-224 datasets demonstrate that features extracted from spatial context provide more information than those from sequence context and the combination of them gives more performance gain. An analysis of the number of binding sites in the spatial context of the target site indicates that the interactions between binding sites next to each other are important for protein-DNA recognition and their binding ability. The comparison between our proposed PDNAsite method and the existing methods indicate that PDNAsite outperforms most of the existing methods and is a useful tool for DNA-binding site identification. A web-server of our predictor (http://hlt.hitsz.edu.cn:8080/PDNAsite/) is made available for free public accessible to the biological research community.

Protein-DNA interactions play important roles in a wide range of fundamental biological processes such as gene regulation, transcription, DNA replication, DNA repair and DNA packaging^1^,^2^,^3^,^4^,^5^. The knowledge about DNA-binding residues, binding specificity and binding affinity helps to not only understand the recognition mechanism of protein-DNA complex, but also give clues for protein function annotation. For example, Ptashne^6^ has reported that the interactions between DNA and transcription factors are essential for gene replication and transcription regulation; Kornberg^7^ has presented that the interactions between DNA and histones are involved in chromosome packaging in the cell nucleus. Bullock and Fersht^8^ have shown that mutations of DNA-binding residues, such as those on the tumor repressor protein P53, may predispose individuals to cancer. Therefore, a reliable identification of DNA-binding sites in DNA-binding protein is important for protein function annotation, in silico modeling of transcription regulation and site-directed mutagenesis. Several experimental techniques have been proposed to identify the DNA-binding sites and investigate the interaction modes between proteins and DNAs. For example, biophysical methods are used to uncover the molecular details of specific residue-residue contacts; alanine-scanning mutagenesis has been employed to identify the amino acids involved in target recognition^9^ by the m5C methyltransferase and to distinguish specific amino acids important for DNA binding and transcription activation by SoxS^10^. However, traditional experimental techniques are very time-consuming and laborious to operate. There is an urgent need for computational tools that can rapidly and reliably identify DNA-binding sites in DNA-binding proteins.

Many machine learning based predictors have been developed for the aforementioned task. They are typically trained from a set of input features, which can be generally divided into three categories: protein sequence information, protein structure information and a combination of the two categories. Protein sequence information mainly consists of amino acid residue composition, biochemical features of amino acid residues and evolutionary information in terms of position-specific scoring matrices (PSSM). Yan and his coworkers^11^ trained a Naïve Bayes classifier by using only sequence information, such as the identities of the target residue and its sequence neighboring residues. Wang and his coworkers^12^ investigated the discriminative power of three sequence features from protein sequence, including the side chain pKa value, the hydrophobicity index and the molecular mass of an amino acid. They then built a SVM classifier for the prediction of DNA-binding sites and constructed a freely accessible web-server BindN. Ofran *et al*.^13^ showed that the DNA-binding residues and the non-binding ones have distinct values in some biophysical characteristics, such as the evolutionary profile, the level of conservation, and the predicted secondary structure and the predicted solvent accessibility. Such characteristics can be used to accurately distinguish the DNA-binding site from non-binding residues. Recently, Wang *et al*.^14^ constructed a DNA-binding site classifier using the evolutionary information in terms of PSSM and several new sequence descriptors including the BLAST-based conservation score, the mean, and the standard deviation of biochemical feature values. Ahmad and his coworkers^15^ developed a DNA-binding site predictor based on Artificial Neural Networks (ANNs) by using only evolutionary information in terms of PSSM. Ho and his coworkers^16^ also used PSSM to train a SVM classifier for the identification of DNA-binding residues. Wang and his coworkers^17^ presented a SVM classifier trained from hybrid features by combining the evolutionary information in terms of multiple sequence alignment and three sequence features. By combining PSSM and four sequence information, Ma *et al*.^18^ developed a SVM classifier for the prediction of DNA-binding sites. They later developed an improved prediction method by proposing two types of novel sequence features and the predictor achieved good performance^19^. These methods apply many helpful sequence information contained in the sequence context for prediction. However, no structural features are used in the site representation.

With the continued accumulation of protein-DNA complex structure data available in Protein Data Bank (PDB)^20^, applying information from the structure of protein-DNA complex to predict DNA-binding sites becomes feasible. In the work done by Ahmad *et al*.^15^, an ANN classifier was developed to distinguish DNA-binding sites from non-binding residues by using the combination of structure information and sequence information. Kuznetsov *et al*.^21^ developed a SVM predictor for the identification of DNA-binding sites by using several categories of structure and sequence information, including PSSM, BLOSUM62, solvent accessibility, and secondary structure. Tjong and his coworkers^22^ constructed a DNA-binding site predictor DISPLAR by training an ANN classifier utilizing solvent accessibility and evolutionary information. A new web-server DR_bind was built recently by Chen and his coworkers^23^. DR_bind was a novel predictor based on electrostatics, evolutionary information and geometry without the need for any training data set. Although these methods presented here have used some important structure information for prediction, there is absence of many helpful sequence features, for example, the evolutionary information and the local amino acid composition.

In fact, most of the existing predictors are trained by hybrid features including both sequence information and structure features. One representative study was by Bhardwaj *et al*.^24^, who combined several categories of information to train a SVM classifier, including residue’s identity, charge, solvent accessibility, average potential, the secondary structure, neighboring residues, and location in a cationic patch. Later, Li *et al*.^25^ developed a classifier by combining machine learning methods and a structural alignment method which detects structural similarity between protein-DNA complexes. They implemented a web-server (PreDNA) for predicting DNA-binding residues. To the best of our knowledge, this is the best-performing predictor up to now.

In conclusion, most methods in the second and the third category mentioned above have used many structure features for prediction, for example, the solvent accessibility, the secondary structure and the amino acid composition of its sequence neighboring residues. However, the information contained in the spatial context are largely ignored. Studies have shown that spatial neighboring residues are important for protein function prediction^22^,^25^, indicating that the spatial context could play an important role in the interaction between protein and DNA molecule. In this study, we first used a spatial sliding window and a sequence sliding window to extract the spatial context and the sequence context for target residues, respectively. Then, the PSSM features, sequence features and structural features in the spatial and the sequence context were extracted to construct the feature space. As the sub-feature space spanned by the PSSM features becomes redundant, Latent Semantic Analysis (LSA) was employed to reduce the dimension of the sub-feature space in order to improve computational efficiency and avoid overfitting. Finally, by integrating SVM and ensemble learning, a predictor, referred to as PDNAsite, for the identification of DNA-binding site was developed. Moreover, a new web-sever at http://hlt.hitsz.edu.cn:8080/PDNAsite/ has been implemented to make PDNAsite freely accessible to the biology research community.

## Materials and Methods

Several recent publications^26^,^27^,^28^ have demonstrated that a useful biological prediction model should consist of valid benchmark dataset(s), an effective feature extraction procedure, an efficient predicting algorithm, a fair evaluation criteria and a web-server. Following this criteria, we describe the PDNAsite in details.

**Figure 1:**
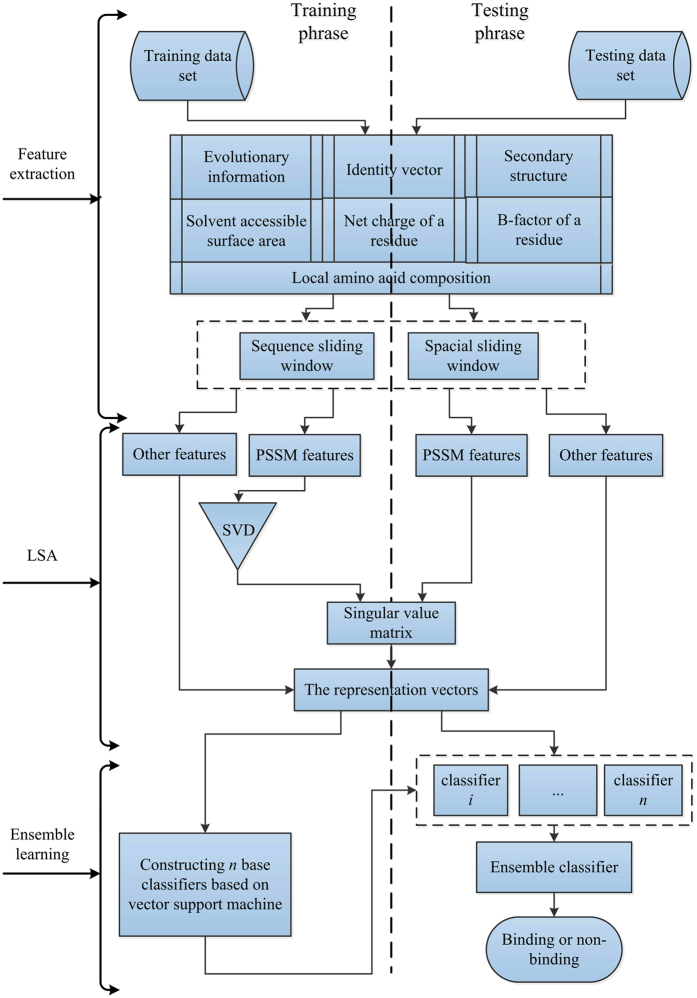
The framework diagram of our proposed PDNAsite. PDNAsite contains three steps: feature extraction, LSA operation and ensemble learning. Feature extraction is used to extracting features from the protein sequence and structure by applying the sequence and spatial sliding windows. LSA operation is applied to the sub feature space spanned by the PSSM features. Ensemble learning is applied to build several base classifiers and then integrates them into an ensemble classier.

The framework diagram of PDNAsite is shown in Fig. 1 where the training phrase is shown on the left panel while the test phase is shown on the right panel. The method contains three steps: feature extraction, LSA operation and ensemble learning. Feature extraction is used to extract seven types of features from the sequence context and the structure context by applying the sequence and spatial sliding windows, respectively. Then LSA is operated on the sub feature space spanned by PSSM features to remove redundancy and reduce dimensionality. Finally, ensemble learning integrates several SVM base classifiers for prediction.

### Data sets

In this study, two protein sequence data sets used in previous studies are used to evaluate the performance of PDNAsite. They are PDNA-62 and PDNA-224, respectively.

#### PDNA-62

PDNA-62 was firstly constructed by Ahmad *et al*.^15^ to train an ANN classifier to distinguish DNA-binding sites from non-binding residues. It was later employed to train different machine learning classifiers by many studies, including ANN, SVM, Random Forest and Naïve Bayes^12^,^14^,^17^,^21^. PDNA-62 was derived from the structure data of 62 protein-DNA complexes in the Protein Data Bank (PDB)^20^. The dataset contains 67 sequences and the sequence identity between any two sequences is less than 25%. As in most previous studies^15^,^17^,^29^,^30^, in the structure of the protein-DNA complexes, a residue in protein is regarded as interacting with DNA if the side chain or the backbone atoms of the residue falls within a cutoff distance of 3.5 Å from any atom of the partner DNA molecule in the complex. All the other residues were regarded as non-binding sites. As a result, this data set contains 1,215 DNA-binding residues and 6,948 non-binding residues. As this dataset has been used in many studies, it is convenient for comparing the predicting accuracy of PDNAsite with that of other existing methods.

#### PDNA-224

PDNA-224 was proposed recently by Li *et al*.^24^ through extracting the structure data of 224 protein-DNA complexes from PDB. It includes 224 protein sequences and the redundancy between any two sequences has been removed by using 25% sequence identity as the cutoff similarity. By using the same criterion as in PDNA-62, there are 3,778 DNA-binding residues and 53,570 non DNA-binding residues. It has been applied to train a DNA-binding site predictor (PreDNA), which is a classifier built by integrating a SVM classifier based on sequence information and a classifier based on structural alignment algorithm. PreDNA has been reported as the best-performing predictor for DNA-binding site prediction up to now. So PDNA-224 was used to evaluate the performance of PDNAsite for comparison with the best-performing predictor. For more detailed information about PDNA-224, please refer to literature^24^.

### Evaluation metrics

In binary-labeled classification problems, five typical evaluation metrics are often used to evaluate the discriminative powers of DNA-binding predictors: Sensitivity (SN), Specificity (SP), Strength (ST), Accuracy (ACC), and Mathews Correlation Coefficient (MCC). The five metrics can be calculated by the following formulae


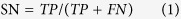



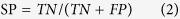



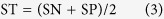



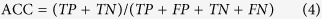



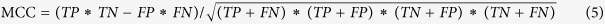


where *TP* is the number of correctly predicted positive instances, *TN* the number of correctly predicted negative instances, *FP* the number of incorrectly predicted negative instances, and *FN* the number of incorrectly predicted positive instances, respectively.

Since the data sets used in this study are imbalanced, the strength(ST), taken as the average of sensitivity and specificity, is used to provide a fair measure of classifier performance^11^,^15^,^30^,^31^. Also, MCC can measure the matching degree between prediction results and real results. Therefore, in this paper, ST and MCC are used as the main metrics and the other three metrics are provided for reference only.

To further evaluate the discriminating power of classifiers on an imbalanced data set, the Receiver Operating Characteristic (ROC) curve^32^ and the area under ROC curve (AUC)^33^ are also used. The ROC curve is probably the most robust approach for classifier evaluation and comparison^33^. The ROC curve is drawn by plotting the true positive rate (i.e. sensitivity) against the false positive rate, which equals to (1-spicificity). In this study, the ROC curve is generated by varying the output threshold of a classifier and plotting the true positive rate against false positive rate for each threshold value. AUC is a reliable measure for classifier performance. An AUC of 1.0 indicates perfect classifier whereas an AUC of classifier no better than random is 0.5.

### Spatial and sequence context

In the study of DNA-binding site prediction, the residue-wise data instances derived from sequence were used as samples to train and evaluate classifiers. In order to make the full use of the sequence context for a target residue, a sliding window of size *w*(being an odd number) is used. Then, a residue-wise data instance was commonly defined as a fragment with *w* consecutive amino acids with the target residue positioned in the middle and (*w*-1)/2 neighboring residues on either side. The residues contained in the sequence sliding window provide the sequence context information for a target residue. However, research results in many literatures^22^,^25^,^34^ have indicated that the spatial context can also contribute to the identification of DNA-binding site from non-binding sites. In order to extract the spatial context of a target site for its prediction, we also proposed a spatial sliding window with size *m*. The spatial sliding window is defined as a sphere with the target site positioned at the center and (*m*-1) sites with the shortest spatial distance to the target site contained in it. The distance between sites is calculated based on the coordinates of their C_α_ atoms.

**Figure 2:**
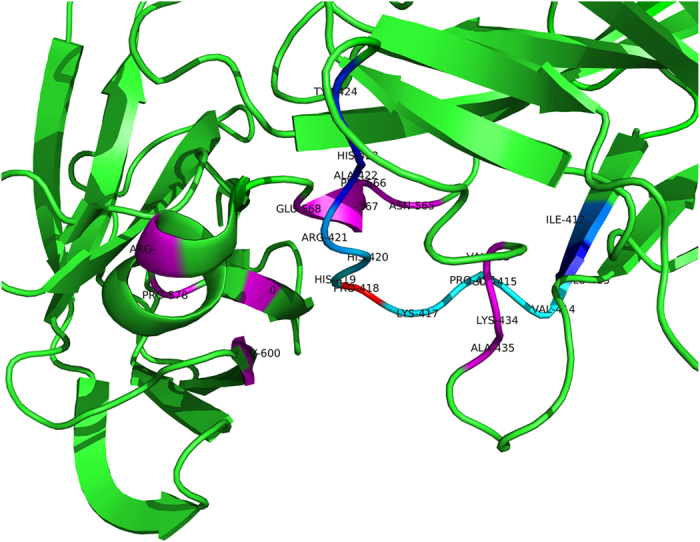
The diagram of the spatial and the sequence context of a site on 1A02. The diagram shows a part of the 3D structure of protein-DNA complex 1A02 and the structure is shown in form of cartoon. The red residue denotes the target site, the combination of the blue and the cyan residues is the sequence context of the target site and the combination of the magenta and the cyan residues is the spatial context of the target site, where the cyan residues are the overlapping sites between the sequence context and the spatial context.

For a target site, the sites contained in the spatial sliding window are referred to as the spatial context, while the sites contained in the sequence window are referred to as the sequence context. As some residues in the sequence context may also be within the cutoff spatial distance from the target site, there may be sites contained simultaneously by both the sequence context and the spatial context, referred to as the overlapping sites. Since these sites are closed to the target site within both the sequence distance and the spatial distance, they can have greater effect on the function of the target site. So when the sequence context and the spatial context are combined to extract features, the overlapping sites should be used twice. Figure 2 shows the diagram of the sequence sliding window and the spatial sliding window of residue P of position 415 in chain N of 1A02. The red residue denotes the target site, the blue and cyan residues are the sites contained in the sequence sliding window and the magenta and cyan residues are the sites contained in the spatial sliding window, where the cyan residues are the overlapping sites between the sequence sliding window and the spatial sliding window.

Therefore, in this paper, a residue-wise data instance is defined as the combination of the sequence context and the spatial context. As a result, a residue-wise data instance should contain (*m* + *w* − 1) residues, since all the overlapping sites apart from the target site should be used twice. A residue-wise data instance is labeled with 1 (positive) if the target residue is binding or −1 (negative) if the target residue is non-binding. As SVM classifiers only take numerical values for classification, the residue-wise data instances need to be encoded into feature vectors. In this work, the feature space of residue-wise data instances is constructed by extracting the sequence information and structure information from the spatial context and the sequence context, including local amino acid composition, evolutionary information in terms of PSSM, B-factor, secondary structure, and Solvent accessible surface area. The spatial context and the sequence context are extracted by applying the spatial sliding window and the sequence sliding window, respectively. Details of the sequence characteristics and the structure characteristics used in this paper will be introduced in the following text.

### Feature extraction

#### Evolutionary information

Evolutionary information in terms of PSSM of protein sequences is obtained by running the PSI-BLAST^35^ program to search against the non-redundant (NR) database through three iterations with 0.001 as the E-value cutoff for multiple sequence alignment. In PSSM, there are 20 values for each sequence position. In order to make full use of the evolutionary information, we process PSSM using the following two steps. First, all elements of a PSSM are scaled between 0 and 1 using the following equation.


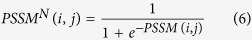


Second, for a residue-wise data instance, the evolutionary information is incorporated into the feature vector by concatenating the corresponding PSSM columns for the sites in the spatial context and the sequence context. Furthermore, for a data instance, the sums in term of evolutionary information for each amino acid type are also calculated for the left sequence sliding window, the right sequence sliding window, the whole sequence sliding window, and the spatial sliding window, and are added into the feature vector.

#### Local amino acid composition

For a data instance, two local amino acid compositions are calculated. One is the amino acid composition for the residues in the sequence sliding window and the other is that for the residues in the spatial sliding window.

#### Identity vector

For a data instance, the identity of the target residue is incorporated by using a 20-feature vector with 1 occurring at the position corresponding to that residue type and 0 for the remaining residue types. For example, if the target residue is Arg, 1 is used at position four with 0 at all other 19 positions.

#### Solvent accessible surface area (ASA)

In this paper, the ASA of every residue in protein is calculated from DSSP^36^. Before encoding the ASAs of the target residue and its neighboring residues, the ASA is divided by the maximum ASA of the corresponding residue type to calculate its relative ASA (RASA). Then, for a data instance, the RASA values of the residues in the spatial context and the sequence context are encoded and added into feature vector.

#### Secondary structure

Secondary structure assignments of all residues in the proteins are made with DSSP^36^, which classify every residue as one of the nine types: alpha helix (H), residue in isolated beta-bridge (B), extended strand participates in beta ladder (E), 3-helix (or 310 helix) (G), 5-helix (or pi-helix) (I), hydrogen-bonded turn (T), bend (S), loop (L) and irregular (no designation). In this paper, the 9 types of secondary structure are approximately combined into 3 types: helix (H), β-strand (E) and coil (C). The secondary structure of the target residue is encoded using mutually orthogonal binary vectors: (1,0,0) for helix, (0,1,0) for β-strand and (0,0,1) for coil. Additionally, the secondary structure compositions for the residues in the left sequence sliding window, the right sequence sliding window, the whole sequence sliding window and the spatial sliding window are added into the feature vector, respectively. The values in the structure composition denote the proportion of the number of residues with the corresponding secondary structure type over the total number.

#### Net charge of a residue

Due to the negative ambience around the DNA, the charge reciprocality of a residue may play an important role in its binding to the partner DNA. Therefore, the net charge of a residue is used as a feature for classification. A charge of +1 is ascribed to Arg and Lys and −1 to Asp and Glu. His is specified a charge of +0.5 and all other residues are taken as neutral. The net charge of the sites in the sequence and spatial sliding windows are calculated.

#### B-factor of a residue

The B-factor of protein crystal structure reflects the fluctuation of atoms about their average positions and provides important information about protein dynamics. The thermal motion is useful for analyzing the dynamic properties of proteins^37^. Therefore, in this work, the B-factor of the C_α_ and that of the C_β_ of the residues in the sequence and spatial windows were encoded. In addition, the sum of the B-factor of the C_α_ over the residues in spatial sliding window was also calculated.

### Latent Semantic Analysis (LSA)

LSA is a method for extracting and representing the contextual meaning of words by statistical computations. Latent Semantic Analysis (LSA) is suitable to remove redundancies in feature space. Recently, LSA has been successfully applied to many bioinformatics problems. For example, Dong and his coworkers^38^ developed SVM classifiers for protein remote homology detection by applying the LSA operation. For this problem, the starting point of the LSA operation is the construction of a triplet-sequence matrix *W* with dimension (M*N) which denotes the co-occurrences between triplets and protein sequences. Triplets denote the combinations of three amino acid types. In the triplet-sequence matrix *W*, each sequence is expressed as a column vector. However, this representation does not recognize the triplets with similar function in the sequence and the dimension is too large. To resolve these problems, singular value decomposition is used to process the triplet-sequence matrix *W*. Let *K* be the rank of *W, W* can be decomposed into three matrices:


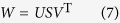


Where *U* is the left singular matrix with dimensions (M*K), *V* is the right singular matrix with dimensions (N*K), *S* is the (K*K) diagonal matrix with singular values where 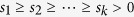. One can reduce the dimensions by deleting the smaller singular values in the diagonal matrix and ignore the corresponding columns of matrix *U* and rows of matrix *V*. Additionally, Liu *et al*.^39^ further improved the prediction accuracy for protein remote homology detection by applying LSA on the top-n-gram-sequence matrix which denotes the co-occurrences between top-n-grams and protein sequences.

Through the analysis of the three matrices (word-document matrix, the triplet-sequence matrix, and the top-n-gram-sequence matrix), we discovered that all these three matrices are constructed by features of the same type, for example, words or biological sequences. We speculate that LSA could only be suitable for processing the feature space constructed by features of the same type, such as words in text, triplet or top-n-gram in protein sequence. In this paper, we construct a feature-instance matrix *W* which denotes the co-occurrences between features and protein sequences. Since there is much redundant information between the PSSM features, we need to apply LSA to decrease the redundant information. However, the features used to construct the matrix *W* do not belong to the same type. So matrix *W* cannot be processed by LSA directly. In this work, we take the sub space of W with dimension of (20*( *w *+*m *− 1)) spanned by only the PSSM features, denoted by *W*′. Since *W*′ contains features of the same, we can then use LSA.

### Support Vector Machine

SVM can be used to resolve both binary-labeled and multi-labeled classification problems. For a binary-labeled classification problem, SVM first maps the input feature space into a higher-dimensional space and then seeks an optimal hyperplane, which maximizes the separation margin between the two classes of training instances, to separate the positive instances from negative instances. As SVM can transform the input features of the instances from a low dimensional space to a higher dimensional space, it has superior generalization power for most classification problems. In this study, the LIBSVM software package available at https://www.csie.ntu.edu.tw/~cjlin/libsvm/40 is used. The radial basis function (RBF) is taken as the kernel function. RBF is defined as


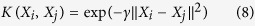


where 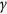 is a training parameter. A smaller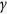 value makes the decision boundary smoother. Another parameter for SVM training is the regularization factor *C*, which controls the trade-off between low training error and large margin. The optimal value of the parameters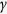 and *C* are obtained by five-fold cross-validation in this work.

The data sets used in this study have many more negative instances than positive instances, which will have a great impact on the prediction performance of classifiers. In order to deal with imbalanced data sets, ensemble learning is used. Ensemble learning first divides the negative instances into *n* folds with non-overlapping instances, where the number of instances in each fold is approximately equal to that of the positive instances. Then the negative instances in each fold and the positive instances are combined to form a new data set. Thus *n* new data sets are constructed. Finally, the *n* new data sets are used as training data sets to train *n* base classifiers, which are subsequently combined as an ensemble classifier for prediction.

Five-fold cross-validation is a widely used validation method, where the data set is first divided into five folds with no overlapping instances, and each time one fold is used as the test set and the remaining four folds are taken as the training set. This process is repeated five times until all the instances in the original set are tested once. The average performance over five such runs is used as the final prediction performance. In this study, the performances of our method on the two data sets are evaluated by applying five-fold cross-validation.

### Performance Results

#### Selection of window sizes *w* and *m*

**Figure 3:**
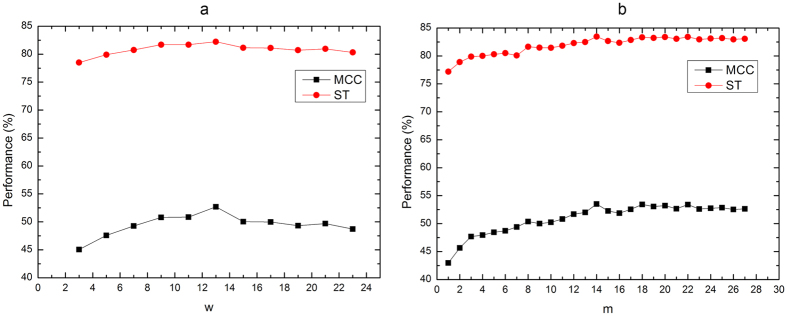
Impacts of window size *w* and *m* on prediction performance. (**a**) The impact of the sequential sliding window size *w* on the prediction performance of PDNAsite; (**b**) The impact of the spatial sliding window size *m* on the prediction performance of PDNAsite

To evaluate the performance of PDNAsite and compare it with other existing predictors, we first analyze the impacts of the sequential sliding window size *w* and the spatial sliding window size *m* on the prediction performance of PDNAsite. The impacts of *w* and *m* on the prediction performance of PDNAsite on PDNA-62 by five-fold cross-validation are shown in Fig. 3a,b, respectively. As can be seen from Fig. 3a, MCC value and ST value are initially on the rise until they reach their maximum value at around w = 13 and then slightly go down with the increasing value of *w*. Thus we choose *w* = 13 for PDNAsite. This value is used for *w* in subsequent analysis. From Fig. 3b, we can see that both MCC and ST values go up as *m* increases and achieves their best values when *m* = 15. So in all the subsequent experiments, *m* is set to 15.

#### Performance comparison of sequence sliding window with spatial sliding window

**Figure 4:**
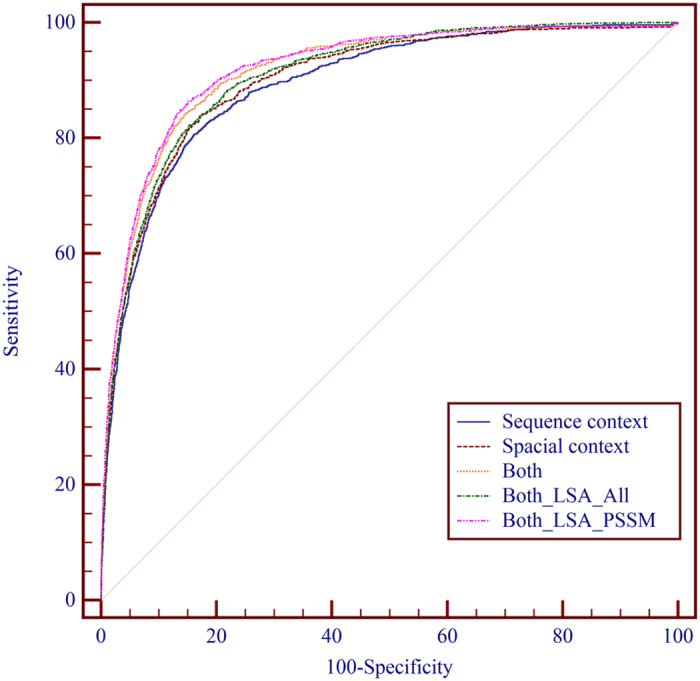
The receiver operating characteristic (ROC) curves of PDNAsite with different sittings on PDNA-62 through five-fold cross-validation.

**Figure 5:**
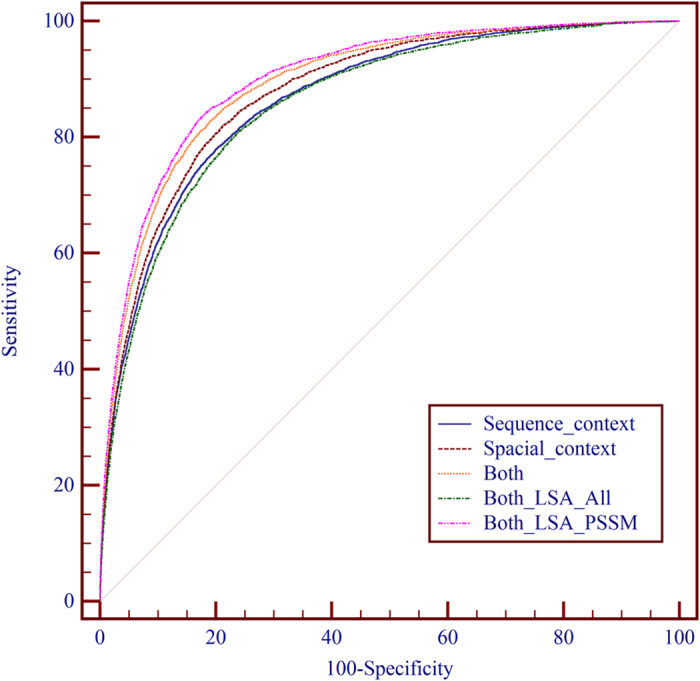
The receiver operating characteristic (ROC) curves of PDNAsite with different sittings on PDNA-224 through five-fold cross-validation.

In this study, the features from the sequence context and the spatial context are used to construct the feature vector for each target site. In order to find out the contributions of the spatial context and the sequence context to the identification of DNA-binding residue, we conduct performance evaluations using three sets of features: sequence context, spatial context and combined use of both. The performances of the predictors using different context on PDNA-62 and PDNA-224 are shown in Tables 1 and 2, respectively. As can be seen from Table 1, on PDNA-62, the predictor using spatial context achieved better performance than that using sequence context by 0.013 in terms of MCC, 0.96% in terms of ST and 0.007 in terms of AUC. The predictor using both of them achieved 0.563 MCC, 84.63% ST and 0.917 AUC, outperforming the one using sequence context alone by 0.036 MCC, 2.41% ST and 0.024 AUC (n = 5, p = 4.15E-4, 1.83E-4 and 3.15E-4, 1-tailed, paired t-test, for MCC, ST and AUC, respectively) indicating the improvement is quite significant. As can be seen from Table 2, on PDNA-224, the predictor using spatial context achieved better performance than that using sequence context by 0.012 MCC, 1.38% ST and 0.012 AUC. The predictor using both of them outperformed the one using sequence context alone by 0.041 MCC, 2.96% ST and 0.026 AUC (n = 5, p = 9.7E-5, 2.54E-4 and 3.1E-5, 1-tailed, paired t-test, for MCC, ST and AUC, respectively) indicating the improvement is quite significant. The ROC curves of the predictors using different contexts on PDNA-62 and PDNA-224 are shown in Figs 4 and 5, respectively. The ROC curves of the predictors with different context also indicate that the spatial context gives more performance gain than the sequence context and the combination of them can further improve the prediction performance.

#### Application of LSA on feature-instance matrix *W*

LSA is an efficient feature extraction technique widely used to remove noise information for a feature space. In this paper, we applied LSA in two different ways: one is applying LSA on the whole feature space, and the other is employing LSA on the sub feature space spanned by PSSM features. The prediction performances of the two ways on PDNA-62 and PDNA-224 by five-fold cross-validation are shown in Tables 3 and 4, respectively. It can be observed that, on PDNA-62, the prediction performance decreased by 0.013 MCC, 1.02% ST and 0.009 AUC when LSA was applied on the whole feature space, while the prediction performance increased by 0.019 MCC, 0.96% ST and 0.005 AUC when LSA was applied on the sub feature space spanned by PSSM features. On PDNA-224, the prediction performance decreased by 0.049 MCC, 3.43% ST and 0.034 AUC when LSA was applied on the whole feature space, while the prediction performance increased by 0.018 MCC, 0.83% ST and 0.008 AUC when LSA was applied on the sub feature space spanned by PSSM features. The ROC curves of the two ways on PDNA-62 and PDNA-224 are shown in Figs 4 and 5, respectively. The results shown in Figs 4 and 5 indicate that LSA is not suitable to deal with the feature space constructed by features of different types and the application of LSA on the sub feature space spanned by PSSM is capable of improving the performance of PDNAsite.

#### Comparison with existing methods

DNA-binding sites have been predicted successfully by many predictors. To demonstrate the discriminating power of our proposed PDNAsite, its prediction performance is compared with other existing state-of-the-art methods. As a meaningful comparison must be made on the same data sets, the following predictors which used the either of the two datasets are used as comparison including Dps-pred^15^, Dbs-pssm^29^, BindN^12^, Dp-bind^21^, Dp-Bind^41^, BindN-RF^14^, BindN+^17^ which used the first dataset, and PreDNA^24^ which used both datasets. PreDNA^24^ is the best-performing predictor reported so far. It integrated a machine learning model and a structural alignment model for prediction where the structural alignment model used the amino acid-nucleotide pairs with distance less than 16 Å as the alignment units. In each alignment unit, the distance between the amino acid and the nucleotide is calculated based on their coordinates in the 3D structure of the protein-DNA complex. However, in most cases, the binding sites and the non-binding sites in the training dataset and the test dataset are defined based on the distances between the sites and its neighboring nucleotides. As such, the binding site can be distinguished from the non-binding site based on the distance information directly. We argue that for training classifiers for DNA-binding sites, the distance information between amino acid and nucleotide should not be used as features. Therefore, in order to fairly compare the performance of our proposed PDNAsite with the existing methods, we only consider the PreDNA without using the structural alignment model.

The prediction accuracies of our method and the existing methods by five-fold cross-validation on PDNA-62 are shown in Table 5. As can be seen from the table, our method performs better than PreDNA by 0.082 MCC, 3.39% ST (n = 5, p = 2.3E-5, 3.36E-4 and 5.0E-5, 1-tailed, one-sample t-test, for MCC, ST and AUC, respectively), indicating that not only PDNAsite is the best performer, the improvement is significant on PDNA-62. The comparison between our predictor and PreDNA^24^ on PDNA-224 by five-fold cross-validation is shown in Table 6. Our method outperforms PreDNA by 0.045 in MCC, 3.47% in ST and 0.010 in AUC (n = 5, p = 1.25E-4, 5.0E-4 and 1.5E-4, 1-tailed, one-sample t-test, for MCC, ST and AUC, respectively), indicating significant performance improvement.

DNABind^42^ is a recently proposed predictor for DNA-binding site prediction, which also used some spatial context as classification features, including degree, closeness and betweenness^43^. These features are calculated from the graph structure formed by the target site and its spatial neighboring sites. The features used in this paper include the amino acid composition, secondary structure, evolutionary information and physiochemical information contained in spatial context. In order to demonstrate the effectiveness of the spatial context proposed in this paper for the prediction of DNA-binding site, we compared our predictor with DNABind^42^ on DS123, HOLO83 and APO83. As our predictor is only trained by DS123 without using any information in the template library used by DNABind^42^, we just compared our predictor with the machine learning-based protocol in DNABind^42^ (DNABind_ML_). The results of the two methods are shown in Table 7. It can be observed that our method outperforms DNABind_ML_ with 2.66% ST and 0.044 AUC (n = 5, p = 8.5E-5, 9.4E-5 and 5.0E-4, 1-tailed, one-sample t-test, for MCC, ST and AUC, respectively) on DS123 and with 3.98% ST and 0.009 AUC on HOLO83. On APO83, our predictor performs similar to that of DNABind_ML_.

DNABR^19^ is a sequence based DNA-binding site prediction method, which performs better than the three methods proposed by Wang *et al*., including BindN, BindN-RF and BindN+. To compare with DNABR, an independent test dataset TS-72 with 72 protein chains is applied. TS-72 was first proposed for evaluating the performance of DNABR^19^ by extracting proteins-DNA complexes from PDB^20^. On the dataset with 3.5 Å as the distance threshold, results show that the AUC values are 0.8783, 0.8669, 0.7488, 8257, and 0.8445 for our method, DNABR, BindN, BindN-RF, and BindN+ method, respectively. For this evaluation our method, BindN, BindN-RF, and BindN+ are trained on the PDNA62 whereas DNABR is trained on a much larger dataset TR265 with 265 protein chains and the AUC values for the other four methods are referenced from Ma *et al*.’s work^19^. It indicates that our method performs better than DNABR and other three methods on TS-72.

#### Analysis of number of binding sites in spatial context

**Figure 6:**
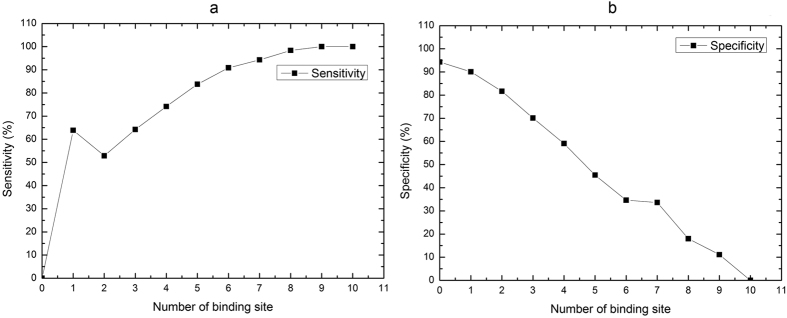
Analysis of number of binding sites in the spatial context. (**a**) The predicting sensitivity of the sites with certain number of binding sites in their spatial context. The x-axis represents the number of binding site contained in the spatial context and the y-axis represents the predicting sensitivity on the sites with certain number of binding sites in their spatial context. (**b**) The predicting specificity of the subset of sites with certain number of binding sites in their spatial context. The x-axis has the same meaning as the one of (**a**) and the y-axis denotes the predicting specificity on the sites with certain number of binding sites in their spatial context.

Different target sites generally have different spatial context. For example, some sites may only contain either non-binding sites or binding sites in their spatial context while other sites may have both of them. Figure 6a,b show the sensitivity and specificity of the predictions for sites with different number of binding sites in their spatial context, respectively. In Fig. 6a, the x-axis represents the number of binding sites contained in the spatial context and the y-axis represents the predicting sensitivity for the sites with certain number of binding sites in their spatial context. In Fig. 6b, the x-axis has the same meaning as the one for Fig. 6a and the y-axis denotes the predicting specificity for the sites with certain number of binding sites in their spatial contexts.

From Fig. 6a, we can see that the predicting sensitivity increases as the number of binding sites in the spatial context increases to 10; and PDNAsite gets the maximal sensitivity when the number of binding sites in the spatial context equals to or greater than 10. From Fig. 6b, we can see that the predicting specificity decreases as the number of binding sites in the spatial context increases to 10; and PDNAsite gets the maximal specificity when the number of binding sites in the spatial context equals to 0. This phenomenon indicates that, as the number of binding sites in the spatial context increases, the target site has more capacity to bind to its corresponding DNA molecule, meaning that the number of binding sites in the spatial context has a great impact on the prediction. Therefore, the spatial context extracted from the spatial sliding window can act as a very important discriminant feature for DNA-binding site identification. We can also conclude that the interactions between neighboring binding sites in their spatial structure are important for protein-DNA recognition and their binding ability.

#### Case study

Epstein-Barr nuclear antigen 1 (PDB 1B3T) activates the initiation of DNA replication once every cell cycle from the Epstein-Barr virus (EBV) latent origin of DNA replication, oriP^44^. Nucleosome Core Particle (PDB 1KX5) is the greater part of nucleosome and comprises an octamer, containing a single histone H3-H4 tetramer and two histone H2A-H2B dimer, and 147 bp of DNA^45^. 1B3T and 1KX5 are two typical protein-DNA complexes and they are not contained by the data sets PDNA-62 and PDNA-224. Moreover, the protein chains in these two complexes show low similarity with that in PDNA-62. So these two complexes are used as study cases for PDNAsite trained on PDNA-62.

**Figure 7:**
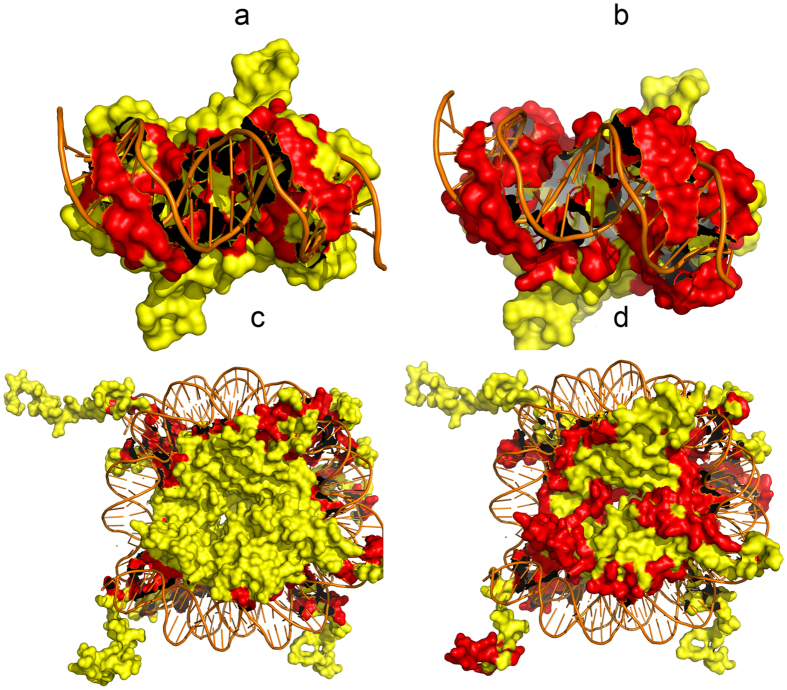
The Real sites and the predicted sites of 1B3T and 1KX5. (**a)** The real sites of 1B3T. (**b**) The predicted sites of 1B3T. (**c**) The real sites of 1KX5. (**d**) The predicted sites of 1KX5. The red region on (**a**,**c**) are the real DNA-binding sites and the red region on (**b**,**d**) are the predicted binding sites by PDNAsite.

On complex 1B3T, PDNAsite achieves 86.16% ACC, 0.599 MCC, 96.00% SN, 84.91% SP and 90.45% ST. And on complex 1KX5, PDNAsite achieves 89.12% ACC, 0.600 MCC, 89.71% SN, 89.06% SP and 89.39% ST. The real DNA-binding sites and predicted sites by PDNAsite for complex 1B3T and 1KX5 are shown in Fig. 7. Figure 7a,b denote the real sites and predicted sites of 1B3T, respectively. And Fig. 7c,d denote the real sites and predicted sites of 1KX5, respectively. From the figure, we can see that most of the real binding sites are covered by the predicted binding sites, indicating that most real binding sites were successfully predicted by PDNAsite. As there are much more non-binding sites than binding sites in a protein sequence, there are some false predicted non-binding sites shown in Fig. 7b,d.

#### Use of the Web-Server of PDNAsite

In this work, we also provides a user-friendly web-server of PDNAsite freely accessible to the public. This section provides a step-by-step guideline on how to use PDNAsite.

##### Step 1

**Figure 8:**
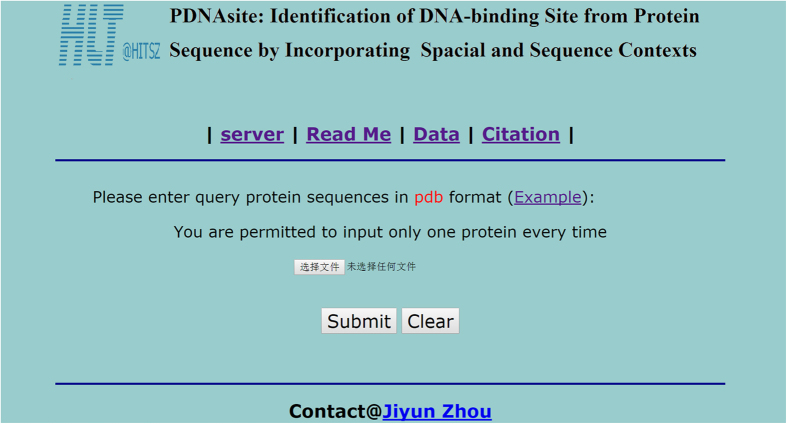
The homepage of the web-server of PDNAsite. The website address of this webserver is http://hlt.hitsz.edu.cn:8080/PDNAsite/.

use the URL (http://hlt.hitsz.edu.cn:8080/PDNAsite/) to get to the homepage of the web-server as shown as in Fig. 8. Click the Read Me button to see instructions on how to use the server.

##### Step 2

Click the Server button for protein sequence query. The input file must be in the PDB format, which contains all the 3D information of the target protein sequence. The input file should be named as the PDB id of the target protein sequence with ‘.pdb’ as the suffix. For example, if the identifier of you target protein sequence is ‘1A02N’, where ‘1A02’ is the PDB id of the protein entry to which the target sequence belongs and ‘N’ is the sequence id of the target sequence, its input PDB file should be name as ‘1A02.pdb’. If the PDB file contains two or more sequences, which is allowed in our system, our server returns the predicting results of all the sequences in the input PDB file.

##### Step 3

**Figure 9:**
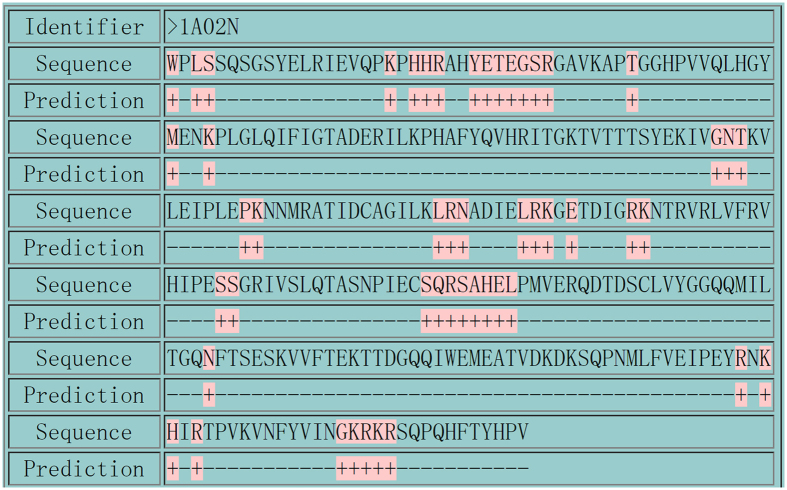
The result page of the web-server of PDNAsite. See the description in the step 3 of the use of the Web-Server of PDNAsite for further explanation.

Once an input file is selected from your file system, click the Submit button to upload your selected file. As the PDNAsite need some time to call some external procedures, such as PSI-BLAST, you need to wait for several minutes until the predicting results are returned to you. Figure 9 demonstrates the predicting results of all the sequences contained in the PDB file of the target protein sequence with identifier as ‘1A02N’, where ‘+’ and ‘−’ represent DNA-binding site and non DNA-binding site, respectively.

## Discussion

A large variety of modern web servers for the prediction of DNA-binding site have been made available for free access to users, including DNABINDPROT^46^, DBindR^47^, BindN^12^, Dp-bind^21^, Dp-Bind^41^, BindN-RF^14^, BindN+^17^, PreDNA^24^, DNABR^19^ and DNABind^42^. DBindR, BindN, Dp-bind, BindN-RF, BindN+ and DNABR are predictors trained by only sequence information, whereas DNABINDPROT, PreDNA, DNABind and our proposed PDNAsite are built by both sequence information and structural information. In general, the methods based on only sequence information have advantage of rapid predicting speed, but their prediction accuracy are lower. For the methods based on the combination of sequence information and structural information, they usually have higher prediction accuracy, but their predicting speed are slower. As DBindR and DNABINDPROT were evaluated on different datasets with PDNAsite, we cannot make quantitative comparisons between PDNAsite and these two predictors. DBindR does not use any protein structure information whereas PDNAsite uses many useful structure features in addition to the features used in DBindR. Thus, we can speculate that PDNAsite can obtain better predicting performance than DBindR. For DNABINDPROT, its SN value is very low. This means that DNABINDPROT will miss many actual DNA binding sites. In contrast, PDNAsite can predict more actual binding sites as candidate DNA-binding sites compared to that of DNABINDPROT. For other methods, the quantitative comparisons on the six datasets demonstrate that PDNAsite performs better than them indicating that our predictor can filter out non-binding residues more precisely and keep more actual DNA binding sites as candidate DNA-binding sites. Therefore, since the experimental determination of DNA-binding site is costly and time-consuming, our web-server can reduce the cost of the experimental methods for DNA-binding site identification by obtaining less false non-binding sites and more actual binding sites as candidate DNA-binding sites and further facilitate the improvement of other bioinformatics problems including DNA-binding protein prediction and the analysis of protein-DNA interactions. Our method is especially useful for biologists who make an attempt to identify the sites involving protein-DNA interaction in protein chains, because we can filter out non-binding residues more precisely and keep the candidate binding residues for further analysis by experiments.

## Conclusion

In this work, spatial sliding window and sequence sliding window are proposed to extract spatial context and sequence context, respectively. Then the features in the spatial and sequence context are combined to construct the feature space. Subsequently, the LSA is applied to reduce the dimension of the feature space. Finally, a predictor (PDNAsite) for the identification of DNA-binding site is developed by integrating SVM classifier and ensemble learning. The prediction performance on the two datasets PDNA-62 and PDNA-224 by five-fold cross-validation demonstrates that: (1) the predictor employing spatial context outperforms the one using sequence context; (2) the predictor employing both context performs better than either of them individually with significant improvement. Consequently, for the identification of DNA-binding site, the spatial context is more significant than the sequence context, and at the same time, both contexts are complementary to each other. Moreover, when LSA is applied to reduce the redundancy in the sub feature space spanned by the PSSM features, the performance of PDNAsite can be further improved. Performance comparisons between PDNAsite and other existing state-of-the-art methods on the datasets demonstrate that our predictor gives the best performance. We have also applied our predictor to predict the binding sites in two typical protein-DNA complexes: 1B3T and 1KX5. The results show that it can predict most of the DNA-binding sites from the protein sequences successfully. An analysis of the number of binding sites in the spatial context for all sites indicates that the spatial context is useful for the identification of DNA-binding site and the interactions between binding sites next to each other are important for the protein-DNA recognition and their binding ability. A web-server of our predictor at http://hlt.hitsz.edu.cn:8080/PDNAsite/ is also made available for free access to the biology research community.

## Additional Information

**How to cite this article**: Zhou, J. *et al*. PDNAsite: Identification of DNA-binding Site from Protein Sequence by Incorporating Spatial and Sequence Context. *Sci. Rep.* **6**, 27653; doi: 10.1038/srep27653 (2016).

## Figures and Tables

**Table 1:** Performance comparison of predictors with different context on PDNA-62 by five-fold cross-validation.

**Methods**	**ACC (%)**	**MCC**	**SN (%)**	**SP (%)**	**ST (%)**	**AUC**
Sequence_context^a^	83.48	0.527	80.40	84.03	82.22	0.893
Spatial_context^b^	83.78	0.540	82.31	84.04	83.18	0.900
Both^c^	84.40	0.563	84.94	84.32	84.63	0.917

^a^The predictor using the sequence context alone.

^b^The predictor using the spatial context alone.

^c^The predictor using both the sequence and the spatial context.

**Table 2:** Performance comparison of predictors with different context on PDNA-224 by five-fold cross-validation.

**Methods**	**ACC (%)**	**MCC**	**SN (%)**	**SP (%)**	**ST (%)**	**AUC**
Sequence_context^a^	79.19	0.346	78.52	79.24	78.88	0.868
Spatial_context^b^	79.61	0.358	81.01	79.50	80.26	0.880
Both^c^	80.81	0.387	83.04	80.63	81.84	0.894

^a^The predictor using the sequence context alone.

^b^The predictor using the spatial context alone.

^c^The predictor using both the sequence and the spatial context.

**Table 3:** Performance comparison of predictor with different LSA on PDNA-62 by five-fold cross-validation.

**Methods**	**ACC (%)**	**MCC**	**SN (%)**	**SP (%)**	**ST (%)**	**AUC**
Both^a^	84.40	0.563	84.94	84.32	84.63	0.917
Both _LSA _All^b^	84.28	0.550	82.63	84.58	83.61	0.908
Both _LSA _PSSM^c^	85.11	0.582	86.27	84.91	85.59	0.928

^a^The predictor using both the sequence and spatial contexts and not applying LSA.

^b^The predictor using both the sequence and spatial contexts and applying LSA on the whole feature space.

^c^The predictor using both the sequence and the spatial context and applying LSA on the sub feature space spanned by the PSSM features, that is, our PDNAsite.

**Table 4:** Performance comparison of predictor with different LSA on PDNA-224 by five-fold cross-validation.

**Methods**	**ACC (%)**	**MCC**	**SN (%)**	**SP (%)**	**ST (%)**	**AUC**
Both^a^	80.81	0.387	83.04	80.63	81.84	0.894
Both_LSA_All^b^	78.54	0.338	78.24	78.57	78.41	0.860
Both_LSA_PSSM^c^	82.25	0.405	83.17	82.34	82.67	0.902

^a^The predictor using both the sequence and the spatial context.

^b^The predictor using both the sequence and the spatial context and applying LSA on the whole feature space.

^c^The predictor using both the sequence and the spatial context and applying LSA on the sub feature space spanned by the PSSM features, that is, our PDNAsite.

**Table 5:** Comparison of PDNAsite with other existing methods on PDNA-62 by five-fold cross-validation^a^.

**Methods**	**ACC (%)**	**MCC**	**SN (%)**	**SP (%)**	**ST (%)**	**AUC**
Dps-pred	79.10	–	40.30	81.80	61.10	–
Dbs-pssm	66.40	–	68.20	66.00	67.10	–
BindN	70.30	–	69.40	70.50	69.95	0.752
Dp-bind	78.10	0.4900	79.20	77.20	78.20	–
DP-Bind	77.20	–	76.40	76.60	76.50	–
BindN-RF	78.20	–	78.10	78.20	78.15	0.861
BindN+	79.00	0.440	77.30	79.30	78.30	0.859
PreDNA	83.06	0.500	80.20	84.10	82.20	–
PDNAsite	85.11	0.582	86.27	84.91	85.59	0.928

^a^The results of Dps-pred^15^, Dbs-pssm^29^, BindN^12^, Dp-bind^21^, Dp-Bind^41^, BindN-RF^14^, BindN+^17^ and PreDNA^24^ and PDNAsite using both the sequence and the spatial context and applying LSA on the sub feature space spanned by the PSSM features.

**Table 6:** Comparison of PDNAsite with PreDNA on PDNA224^a^ by five-fold cross-validation.

**Methods**	**ACC (%)**	**MCC**	**SN (%)**	**SP (%)**	**ST (%)**	**AUC**
PreDNA	81.80	0.350	76.10	82.20	79.20	0.892
PDNAsite	82.25	0.405	83.17	82.34	82.67	0.902

^a^The results of PreDNA^24^ and PDNAsite using both the sequence and the spatial context and applying LSA on the sub feature space spanned by the PSSM features.

**Table 7:** Comparison of PDNAsite with DNABind on DS123, HOLO83 and APO83.

**Datasets**	**Methods**	**ACC (%)**	**MCC**	**SN (%)**	**SP (%)**	**ST (%)**	**AUC**
DS123	DNABind^a^	80.76	0.432	69.80	82.76	76.28	0.845
	PDNAsite^b^	84.56	0.506	71.02	86.86	78.94	0.889
HOLO83	DNABind^a^	83.25	0.411	59.00	87.09	73.05	0.839
	PDNAsite^b^	78.66	0.439	74.59	79.47	77.03	0.848
APO83	DNABind^a^	83.47	0.396	58.30	87.36	72.58	0.837
	PDNAsite^b^	77.18	0.373	65.18	78.92	72.07	0.829

^a^Denotes the machine learning-based protocol in DNABind^42^.

^b^Denotes the PDNAsite using both the sequence and the spatial context and applying LSA on the sub feature space spanned by the PSSM features.
